# Sulfite Oxidase Activity Is Essential for Normal Sulfur, Nitrogen and Carbon Metabolism in Tomato Leaves

**DOI:** 10.3390/plants4030573

**Published:** 2015-08-14

**Authors:** Galina Brychkova, Dmitry Yarmolinsky, Albert Batushansky, Vladislav Grishkevich, Inna Khozin-Goldberg, Aaron Fait, Rachel Amir, Robert Fluhr, Moshe Sagi

**Affiliations:** 1 French Associates Institute for Agriculture and Biotechnology of Drylands, Blaustein Institutes for Desert Research, Ben-Gurion University of the Negev, Sede Boqer Campus 84990, Israel; E-Mails: galina.brychkova@nuigalway.ie (G.B.); dmitry.yarmolinsky@ut.ee (D.Y.); albert.batushansky@gmail.com (A.B.); grivvol@gmail.com (V.G.); khozin@bgu.ac.il (I.K.-G.); fait@bgu.ac.il (A.F.); 2 Migal-Galilee Technology Center, Southern Industrial Zone, POB831 Kiryat-Shmona 11016, Israel; E-Mail: rachel@migal.org.il; 3 Department of Plant Sciences, Weizmann Institute of Science, P.O.B. 26 Rehovot 76100, Israel; E-Mail: robert.fluhr@weizmann.ac.il

**Keywords:** sulfite oxidase, dark-induced senescence, sulfur, nitrogen, carbon metabolism, lipid degradation

## Abstract

Plant sulfite oxidase [SO; E.C.1.8.3.1] has been shown to be a key player in protecting plants against exogenous toxic sulfite. Recently we showed that SO activity is essential to cope with rising dark-induced endogenous sulfite levels in tomato plants (*Lycopersicon esculentum/Solanum lycopersicum* Mill*. cv. Rheinlands Ruhm*). Here we uncover the ramifications of SO impairment on carbon, nitrogen and sulfur (S) metabolites. Current analysis of the wild-type and SO-impaired plants revealed that under controlled conditions, the imbalanced sulfite level resulting from SO impairment conferred a metabolic shift towards elevated reduced S-compounds, namely sulfide, S-amino acids (S-AA), Co-A and acetyl-CoA, followed by non-S-AA, nitrogen and carbon metabolite enhancement, including polar lipids. Exposing plants to dark-induced carbon starvation resulted in a higher degradation of S-compounds, total AA, carbohydrates, polar lipids and total RNA in the mutant plants. Significantly, a failure to balance the carbon backbones was evident in the mutants, indicated by an increase in tricarboxylic acid cycle (TCA) cycle intermediates, whereas a decrease was shown in stressed wild-type plants. These results indicate that the role of SO is not limited to a rescue reaction under elevated sulfite, but SO is a key player in maintaining optimal carbon, nitrogen and sulfur metabolism in tomato plants.

## 1. Introduction

It is commonly assumed that sulfur (S), carbon (C) and nitrogen (N) pathways should be well coordinated in order to maintain the production of S-amino acids in plants [1]. However, C:N:S ratio disruption could also lead to alterations of other metabolic processes, as shown with sulfur starvation that affects plant nitrogen metabolism [2], and impairment in sulfite reductase activity which results in amendment of nitrogen and carbon metabolisms [3]. Catabolic processes induced by nutrient deprivation and senescence result in plant metabolism rebalancing [2,4]. These processes include a decrease in cellular carbohydrate levels and respiration rate, breakdown of proteins, membrane lipids, nucleic acids, polysaccharides, chlorophyll and other complex organic molecules [5,6,7]. Carbon starvation-induced senescence is also associated with a increase in free amino acids [8,9,10,11], which could either be a result of protein degradation, or of *de-novo* amino acid biosynthesis. Another hallmark of senescence is the enhancement of substances with high N/C ratios, such as ureides, allantoin and allantoate [12], as well as amides, which are preferable storage compounds from an energetic point of view [13]. To delay senescence during accelerated catabolism, the over-accumulation of toxic by-products such as ammonia, sulfite, sulfide, reactive oxygen species (ROS) and others, should be prevented by the activation of detoxification/re-assimilation processes [12,14,15]. 

Sulfite oxidase [SO; Enzyme Commission (EC) 1.8.3.1] is crucial for degradation of sulfur-containing compounds in animals and plants [14,16,17,18,19,20,21,22,23,24]. Plant SO is a molybdenum cofactor (MoCo)-containing enzyme, localized in the peroxisomes, which catalyzes the oxidation of sulfite to sulfate [25]. Sulfite is a toxic nucleophile the level of which must be carefully controlled. SO deficiency is not necessarily lethal, unless other sulfite network enzymes are down-regulated [14] or the capacity of the sulfite network enzymes in sulfite detoxification is exceeded [14,19,26]. In plants, alternative sulfite-scavenging systems include the major metabolic sink, the chloroplast-localized sulfite reductase (SiR; EC 1.8.7.1). Recently we showed that SiR plays an important role in prevention of premature senescence as a result of sulfite overproduction by enhanced APR (adenosine-5′-phosphosulfate (APS) reductase) activity [15]. Sulfite levels can also be regulated by being incorporated into sulfolipids, catalyzed by the chloroplast-localized UDP-sulfoquinovose synthase (SQD1; EC 3.13.1.1) or can be converted to the less toxic compound thiosulfate catalyzed by sulfurtransferases (STs; EC 2.8.1.2.) [14]. The STs are a large protein family with members localized in the cytosol and cell organelles such as the chloroplast, mitochondrion and nucleus [27]. Another member of the sulfite network is the chloroplast-localized APR (EC 1.8.4.9) that catalyzes the generation of sulfite from sulfate taken up by the roots and translocated to the leaves [14]. We and others have previously shown that SO-deficient plants are more susceptible to exogenously applied high concentrations of sulfite [14,19,21,23]. To demonstrate the role of SO in the oxidation of endogenously generated sulfite, we recently employed a successive dark-induced senescence for 11 days as an experimental platform to induce accelerated catabolism and generate differences in the response of the SO mutants as compared to the wild-type plants [14]. The extended dark-induced senescence methodology is widely used, even when applied for 10 to 15 successive days [28,29,30,31,32,33,34], as a model to investigate metabolic homeostasis altered as a result of certain enzyme attenuation. We showed that, in the wild-type plants, SO expression was upregulated by the extended dark period, while the expression of the other sulfite network components, APRs, SiR, SQD1 and STs, was inhibited. In contrast, SO impairment in the mutant plants caused the accumulation of sulfite at toxic levels as a result of the dark-induced S-containing metabolite degradation, leading to increased leaf damage and plant mortality (30% to 40% of the mutants survived, *vs.* 90% survival rate among the dark-stressed wild-type plants). These results show that SO activity is necessary to cope with rising endogenous sulfite levels [14]. However, the ramifications of impairment in SO activity on the carbon (C), nitrogen (N) and additional important sulfur (S) metabolites have not previously been shown.

By analyzing the C, N and S metabolism in samples collected from the same extended dark stress and unstressed plants as described by us before [14], we show here that the role of SO is not limited to that of a rescue reaction under elevated sulfite levels, but SO is a central player in the mediation of primary metabolism. The absence of active SO resulted in a significant elevation of the levels of reduced sulfur-type compounds such as sulfide, S-containing amino acids, Co-A and acetyl-CoA. The enhancement of S-amino acids was accompanied by the enhancement of total non-S amino acids, as well as N and C metabolites, including total polar lipids. Exposing the plants to dark stress resulted in a higher degradation rate of S-containing metabolites, carbohydrates, polar lipids, total amino acids and RNA in the mutant plants. The results indicate that SO activity is essential to maintaining an optimal C, N and S metabolism in dark-stressed and unstressed tomato plants.

## 2. Results and Discussion 

### 2.1. Results

A significant reduction of total plant biomass was noticed in the mutant plants as compared to the wild-type plants grown under normal unstressed growth condition, although no obvious tissue damage and enhanced expression of stress marker genes were noticed in the mutant plants, (Supplementary Figure S1). We hypothesize that active SO not only contributes to sulfite detoxification [14,19,21,23], but also to the homeostasis of the sulfate reduction pathway, and that through this function it may impact N, C and S metabolisms. To examine this hypothesis, tomato wild-type (*Lycopersicon esculentum/Solanum lycopersicum* Mill*. cv. Rheinlands Ruhm*) and plants impaired in SO activity by RNA interference (Ri), employed for the transcript and protein analysis described by Brychkova *et al.*, [14] were used here as the basis for global metabolic profiling. A mix of top leaves (fifth and sixth from the bottom (see Experimental Section)) of six-week old tomato plants was collected before the dark treatment (T0) and from surviving plants after 11 days of growth in the dark (T_11_). The eleven days dark stress resulted in a relatively low increase of total biomass (0.28 and 0.27-fold enhancement in WT and SO Ri plants, respectively (Supplementary Figure S1a)) unlike the plants grown under normal day/night conditions for the same period of time. Indeed, due to the indeterminate growth pattern under normal growth conditions tomato plants demonstrated a total biomass increase of 2.1 and 2.33-fold in WT and SO Ri plants, respectively, resulting in the growth of additional three leaves. Employing T_0_ as control to the T_11_ dark-treated plants enabled us to sample the same top leaves in both T0 and T11 plants, still using the fifth and sixth leaves from the bottom.

Principal component analysis (PCA) of 77 metabolites was employed to obtain a global view of the effect of plant genotypes and treatments on plant metabolism. An integrated data set was obtained by median normalization of the data (Supplementary Table S1) generated by different analytical platforms (See Experimental Section, Supplementary Figure S2) [35,36].

The dispersion of the samples over the two first components on the plane suggests a different metabolic response of all samples to dark stress (0 (normal growth) *vs.* 11 days under dark stress conditions), as well as a genotype effect (wild-type *vs.* SO Ri plants). The distribution of samples across the first principal component (PC_1_, Supplementary Figure S2, X-axis), which accounts for 59.9% of the total variance, generates distinct groups underlying differences in metabolism in respect to dark-induced senescence *vs.* normal growth conditions. The second principal component (PC_2_) accounts for 20.8% of the total data variance (Supplementary Figure S2, Y-axis) and it discriminates samples according to their genotypes. The effect of genotype was pronounced mainly in response to the dark treatment (right upper and lower quadrants), yet, under normal growth conditions both the wild-type and the SO Ri mutants showed a degree of segregation as well (left upper quadrant). Thus, the metabolite composition was genotype-dependent, and it was relevant for examining the metabolic alteration in major S, C and N metabolites in the wild-type and SO Ri mutants grown under normal growth conditions and after extended dark stress.

#### 2.1.1. The Effect of SO Impairment on the Metabolism of S-Containing Metabolites

**Figure 1:**
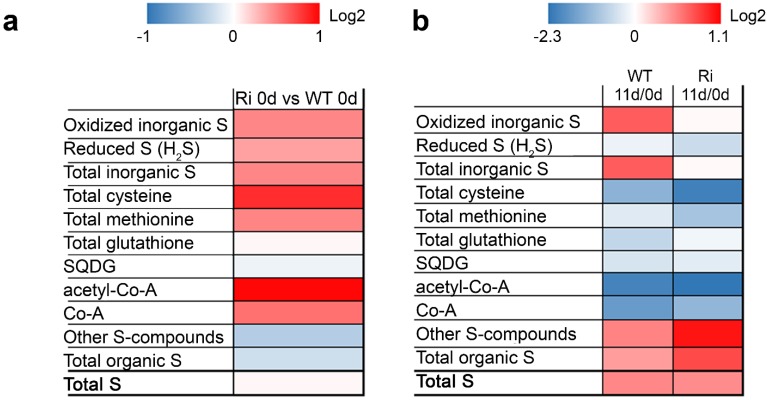
Turnover of sulfur containing metabolites in wild-type (Wt) and sulfite oxidase (SO) RNA interference mutants (Ri) plants grown under normal growth conditions (0 day) and after being exposed to an extended dark stress for eleven days (11 day). The Log2 of sulfur containing metabolites are given by shades of red or blue colors according to scale bar. (**a**) Relative difference of sulfur-containing metabolites in SO Ri mutants calculated as ratio to WT 0 day; (**b**) The relative difference of S-containing metabolites was calculated as the ratio of metabolite content in plants after the dark stress (11 day) to the content detected in the unstressed plants (0 day). The other S-compounds metabolites were calculated as the difference between total content of sulfur to the known S-containing metabolites. The organic S was calculated as the difference between total S to the inorganic S (oxidized + reduced). The level of significance was estimated with Turkey-Kramer HSD test (JMP 8.0 software, [37]; *p* < 0.05. *n* = 3–10) and is presented in Supplementary Table S2. The data for SO-compromised plants represent the mean for SO Ri 131 and SO Ri 421 mutants.

Under normal growth conditions, the total sulfur content of both genotypes is similar, yet its distribution differs remarkably (Figure 1a). The levels of inorganic oxidized sulfur compounds, *i.e.*, sulfate, sulfite and thiosulfate, as well as the reduced sulfur in the sulfide form, total S-amino acids, Co-A and acetyl-Co-A were significantly increased in the SO Ri mutants as compared to the wild-type (Figure 1a; Supplementary Table S2). The difference between total S and inorganic S is defined as organic sulfur and includes the thiols, Cys and Met, all the coenzymes and the secondary S-containing metabolites. Consequently, under normal unstressed conditions the total organic sulfur content was significantly lower in SO mutants as compared to wild type plants (Figure 1a). Nevertheless, cysteine, methionine, coenzyme-A and acetyl-Co-A were elevated in SO Ri mutants compared to the WT plants grown under normal conditions (Figure 1a). It is likely that in the absence of active SO the essentiality to detoxify the generated sulfite, especially in the vicinity of the chloroplast, leads to a metabolic shift towards higher production of the reduced sulfur-type metabolites sulfide, Cys and Met (Figure 1), which is in agreement with the higher activity of the chloroplast localized SiR enzyme in SO mutants [14].

The extended dark stress resulted in a significant enhancement of the oxidized S-compounds in the wild-type plants, while in the SO-impaired plants the total amount of oxidized S-compounds (other than sulfite) was unchanged (Figure 1 and [14]). The total organic sulfur was elevated in response to dark stress in both genotypes, although the total Cys and Met were significantly decreased in mutants in comparison to the wild-type plants (Figure 1b; Supplementary Table S2; The degraded S-metabolites were calculated as the difference between metabolite content in the unstressed plants and the content detected in plants after dark stress). In response to dark stress total glutathione was decreased by about 1/3 in the wild-type plants but only by ~1/10 in the mutants. Stress-induced consumption of glutathione has been documented [23] and appears to be impaired in the mutants. This defect may be correlated with the increased ROS levels that were up to two-fold higher in the SO Ri mutants under all conditions as compared to the wild-type levels (Supplementary Figure S3). Dark stress also resulted in a decrease in the level of sulfoquinovosyl diacylglycerols (SQDG) in both the wild-type and mutant plants (Figure 1, Supplementary Table S2). In contrast, the amount of degraded CoA and acetyl-CoA in the SO Ri mutants was significantly higher compared to the wild-type plants exposed to dark stress (Supplementary Table S2). The modification in the CoA levels is indicative of possible metabolic changes in fatty acid biosynthesis and in the tricarboxylic acid cycle (TCA cycle), where CoA and its derivatives play a central role.

#### 2.1.2. SO Mutation Affects Carbon Metabolism in Normal and Dark-Stressed Plants

**Figure 2:**
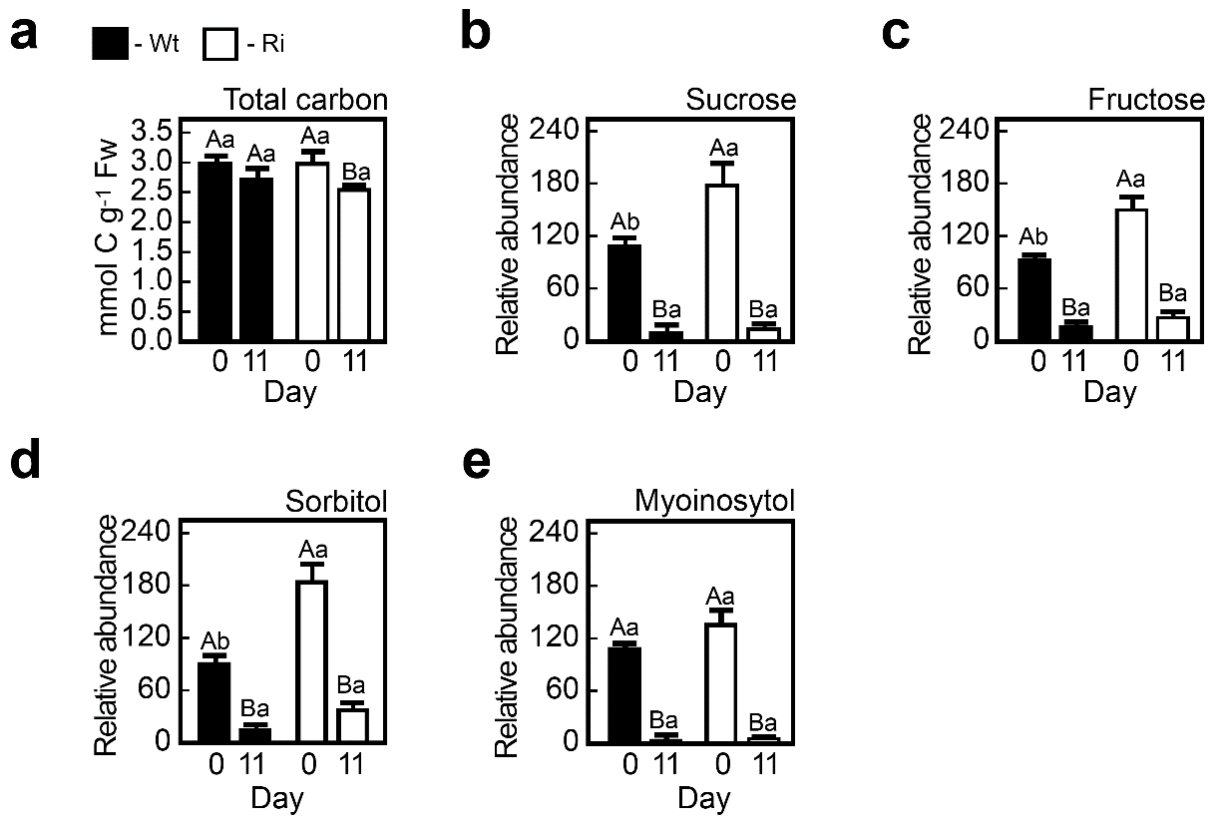
Energy-source metabolites in wild-type (Wt) and SO RNA interference mutants (Ri) plants grown under normal growth conditions (0 day) and after being exposed to eleven days of dark stress (11 day). Top leaves of WT and Ri tomato plants were used to determine total carbon levels (**a**). Relative carbohydrate, sugars sucrose (**b**); fructose (**c**); sugar alcohols; sorbitol (**d**) and myoinositol (**e**) contents were also determined. The bars represent the average values ± SE (*n* = 3–8). Values denoted with different letters are significantly different according to the Turkey-Kramer HSD test (JMP 8.0 software, [37]; *p* < 0.05). Different lower case letters indicate differences between the SO mutants and wild-type plants under the same treatment. Different upper case letters indicate significant differences within the plant genotypes in response to treatment. The data for the SO-compromised plants represent the mean for SO Ri 131 and SO Ri 421 mutants.

Normally grown wild-type and SO Ri mutants did not significantly differ in their total carbon content, detected by elemental analyzer (Figure 2a). However, individual sugars such as sucrose and fructose (Figure 2b,c) and sugar alcohol sorbitol (Figure 2d) in the SO Ri mutants were significantly elevated under normal growth conditions compared to the wild-type plants (Figure 2). 

Interestingly, these differences disappeared under the stress treatment. The measurements indicate that under normal growth conditions impairment in SO does not affect the total carbon level but rather modifies the carbon profiles. Under the dark stress conditions these differences disappeared with the enhanced sugar and sugar alcohol consumption as compared to the wild-type plants. 

**Figure 3:**
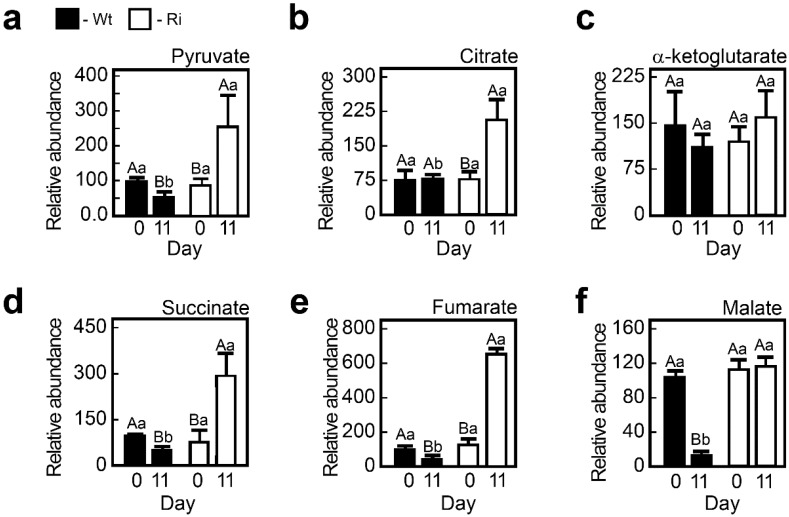
The levels of pyruvate, the product of glycolysis, and tricarboxylic acid cycle (TCA) cycle intermediates in wild-type (Wt) and SO RNA interference mutants (Ri) plants grown under normal growth conditions (0, day) and after being exposed to eleven days of extended dark stress (11, day). Top leaves of Wt and Ri tomato plants were used to determine pyruvate (**a**); citrate (**b**); a-ketoglutarate (**c**); succinate (**d**); fumarate (**e**) and malate (**f**) content. The bars are the average values ± SE (*n* = 3–8). The values denoted with different letters are significantly different according to the Turkey-Kramer HSD test (JMP 8.0 software, [37]; *p* < 0.05). Different lower case letters indicate differences between the SO mutants and wild-type plants within the same treatment. Different upper case letters indicate significant difference within the plant genotypes in response to treatment. The data for SO-compromised plants represent the mean for SO Ri 131 and SO Ri 421 mutants.

Glycolysis and the TCA cycle pathways are central metabolic pathways for the production of ATP and reductants, as well as a source for carbon skeletons for the biosynthesis of macromolecules. Under normal growth conditions, no significant differences were noted between the wild-type and SO-impaired plants in the levels of key metabolites, *i.e.*, pyruvate as the glycolysis product, and in the levels of the TCA cycle intermediates such as citrate, α-ketoglutarate, succinate, fumarate and malate (Figure 3a–f). However, under extended dark stress major differences were found between the two genotypes. In the mutants, levels of pyruvate, citrate, succinate, malate and fumarate were elevated by two to six-fold compared to the dark-stressed wild-type leaves (Figure 3). Only α-ketoglutarate levels were similar in the mutants and wild-type under both studied conditions.

The dark-induced elevation of the pyruvate level, as well as the levels of nearly all measured TCA metabolites in the SO mutants, may indicate impairment in the TCA cycle activity or in cataplerotic reactions of the cycle for *de-novo* biosynthesis of amino acids, lipids and others. Such a synthesis has been noted after feeding low concentrations of the sulfhydryl reagent dithiothreitol, indicating the fundamental pleiotropic effects of the thiol status on plant metabolism [38].

#### 2.1.3. SO Mutation Alters Lipid Metabolism under Normal and Dark-Stressed Conditions

**Figure 4:**
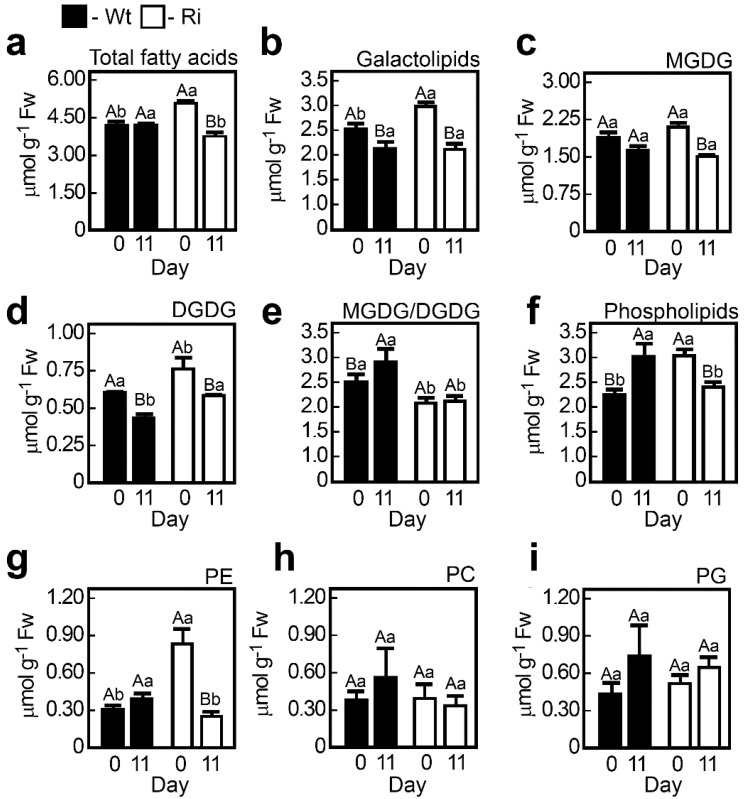
Lipid content in tomato wild-type (Wt) and SO RNA interference mutant (Ri) plants grown under normal growth conditions (0, day) and after being exposed to eleven days extended dark stress (11, day). Total polar lipids (**a**), total galactolipids (**b**) and individual galactolipids, (**c**,**d**) as well as the ratio between the galactolipids (**e**), total phospholipids (**f**) and individual phospholipids (**g**–**i**) were quantified by Trace GC Ultra as their methyl esters of the constituent fatty acids after two-dimensional thin-layer chromatography. First dimension was chlorophorm:Methanol:H_2_O (65:25:4, v/v/v) followed byChlorophorm:Methanol:NH_4_OH: Isopropylamine (65:35:5:0.5, v/v/v/v) and visualization under UV. Abbreviations are: digalactosyl-diacylglycerol (DGDG); monogalactosyl-diacylglycerol (MGDG); phosphatidylglycerol (PG); phosphatidylcholine (PC); phosphatidylethanolamine (PE). The bars are the average values ± SE (*n* = 3). The values denoted with different letters are significantly different according to the Turkey-Kramer HSD test (JMP 8.0 software, [37]; *p* < 0.05). Different lower case letters indicate differences between SO mutant and wild-type plants within the same treatment. Different upper case letters indicate significant difference within the plant genotypes in response to treatment. The data for SO-compromised plants represent the mean for SO Ri 131 and SO Ri 421 mutants.

Lipid content in plants is crucial for plant health [39,40]. Leaf senescence triggers intensive catabolism of membrane lipids, primarily targeting chloroplasts [41]. Using the method previously described by us [42], we detected ca. 20% total fatty acids enhancement in the SO Ri mutants as compared to the wild-type plants grown under normal unstressed conditions (Figure 4a). Dark stress results in higher lipid content reduction in the SO Ri mutants, making the total fatty acid level comparable to that in the wild-type plants (Figure 4). 

This is evident in the major lipid fraction of the chloroplast thylakoids, the galactolipids monogalactosyldiacylglycerol (MGDG) and digalactosyldiacylglycerol (DGDG, Figure 4b–d), synthesized in the chloroplast envelope [43,44,45]. In the unstressed SO Ri mutant plants, the levels of DGDG but not those of MGDG were enhanced as compared to the wild-type plants. Extended dark stress resulted in the decrease of DGDG in both genotypes and the decrease of MGDG only in the mutants, while no significant effect was evident in the wild-type plants (Figure 4c–e).

The levels of phospholipids, phosphatidylcholine (PC) and phosphatidylglycerol (PG), did not differ much between the genotypes and were not affected by the extended dark stress (Figure 4f,h,i). Exceptionally, phosphatidylethanolamine (PE) levels were more than two-fold higher in the SO Ri plants, however, after 11 day of dark treatment a reduction by a third of PE was noted in the mutants (Figure 4g). These results indicate that the absence of active SO alters polar lipid metabolism in unstressed plants and modifies polar lipid catabolism in the dark-stressed plants.

Under unstressed conditions, only dienoic linoleic acid (18:2 *n*-6) was significantly reduced in the mutants as compared to the unstressed wild-type plants. Dark stress resulted in a significant enhancement of stearic acid (18:0) and unsaturated α-linolenic acid (18:3 *n*-3) in both genotypes, as well as a significant enhancement in the proportion of unsaturated hexadecatrienoic acid (16:3 *n*-3) in the mutants but not in the wild-type plants. A significant decrease was evident in the proportion of the unsaturated oleic acid (18:1 *n*-9) and linoleic acid (18:2 *n*-6) in both genotypes (Table 1).

Sulfolipids sulfoquinovosyl diacylglycerols (SQDG) biosynthesis is dependent on the reductive sulfate assimilation pathway. While the total SQDG levels under normal conditions were similar in both genotypes (Table 1), the SQDG profile revealed a significant increase in the levels of unsaturated linoleic acid (18:2 *n*-6) and α-linolenic acid (18:3 *n*-3), as well as saturated palmitic acid (16:0) in the SO-impaired mutants as compared to the unstressed wild-type plants (Table 1). In contrast, the proportions of 18:1 *n*-9 and 18:0 were decreased. As for the response to dark stress, SQDG exhibited a significant enhancement of 18:3 n-3 and 16:0 and a decrease in the proportion of stearic acid (18:0), oleic acid (18:1 *n*-9) and unsaturated linoleic acid (18:2 *n*-6) in both genotypes (Table 1). These results indicate that SO mutation broadly affects lipid metabolism under normal and extended dark stress conditions. 

**Table 1:** Fatty acid composition (% of total) of total polar lipids (PL) and SQDG in wild-type (Wt) and SO RNA interference mutants (Ri) plants grown under normal growth conditions (0 day) and after being exposed to eleven days of extended dark stress (11 day) *.

Lipids	Genotype	Time in Dark (d)	16:0	16:1	16:1^Δ3t^	16:2	16:3 *n*-3	18:0	18:1 *n*-9	18:2 *n*-6	18:3 *n*-3
PL	Wt	0	20.1 Aa	2.1 Aa	1.4 Aa	0.6 Aa	6.0 Aa	2.9 Ba	2.6 Aa	25.2 Aa	39.1 Ba
		11	21.4 Aa	3.7 Aa	1.2 Ab	0.7 Aa	6.4 Ab	4.0 Aa	1.4 Ba	14.9 Ba	46.3 Aa
	Ri	0	19.3 Aa	2.8 Aa	1.6 Aa	0.7 Aa	6.4 Ba	2.7 Ba	3.0 Aa	22.0 Ab	41.5 Ba
		11	19.4 Aa	4.0 Aa	1.9 Aa	0.5 Aa	7.9 Aa	3.3 Ab	1.5 Ba	13.6 Ba	47.9 Aa
SQDG	Wt	0	39.5 Bb	2.1 Aa	--------	0.4 Aa	0.7 Aa	9.4 Aa	18.9 Aa	9.2 Ab	19.8 Bb
		11	43.5 Aa	1.3 Aa	--------	0.1 Aa	0.9 Aa	5.4 Ba	11.7 Ba	7.6 Bb	29.5 Aa
	Ri	0	42.8 Ba	1.9 Aa	--------	0.1 Aa	0.6 Aa	4.6 Ab	8.7 Ab	17.5 Aa	23.8 Ba
		11	46.5 Aa	3.1 Aa	--------	0.0 Aa	0.7 Aa	3.8 Ba	1.9 Bb	12.5 Aa	31.5 Aa

*. Abbreviations are: Sulfoquinovosyl diacylglycerols (SQDG); RNA interference (Ri). The values denoted with different letters are significantly different according to the Turkey-Kramer HSD test (JMP 8.0 software, [37]; *p* < 0.05, (*n* = 3 experiments). Different lower case letters indicate differences between SO mutant and wild-type plants at the same treatment. Different upper case letters indicate significant differences within the plant genotypes in response to treatment. The data for SO-compromised plants represent the mean for SO Ri 131 and SO Ri 421 mutants.

#### 2.1.4. Effect of SO Silencing on Nitrogen Metabolism and Amino Acids Composition

**Figure 5:**
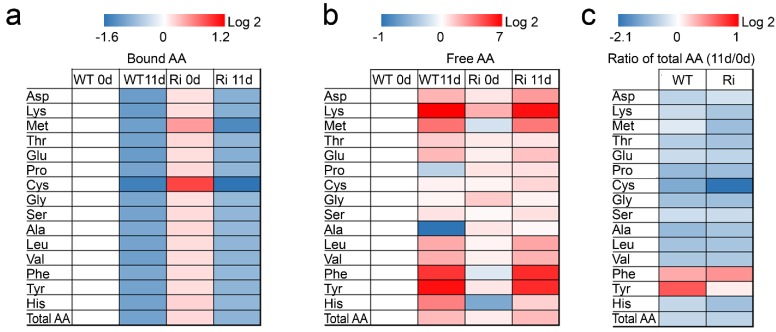
Heat map of free and protein bound amino acids in wild-type (WT) and SO RNA interference mutant (Ri) plants grown under normal growth conditions (0, day) and after being exposed to eleven days of extended dark stress (11, day). Log2 ratios of bound (**a**) and free (**b**) amino acids (AA) fold-changes from WT 0 day are given by shades of red or blue colors according to the scale bar; (**c**) Log2 ratios of residual to initial total AA were calculated as the Log2 ratio of total AA at 11 day to 0 day and are given by shades of red or blue colors according to scale bar. Asp—aspartate; Lys—lysine; Met—methionine; Thr—threonine; Glu—glutamate; Pro—proline; Cys—cysteine; Gly—glycine; Ser—serine; Ala—alanine; Leu—leucine; Val—valine; Phe—phenylalanine; Tyr—tyrosine; His—histidine. Data represent the mean value of three (WT) and six (Ri) biological replicates for each tissue and time point and statistical analysis performed using Turkey-Kramer HSD test (JMP 8.0 software, [37]; *p* < 0.05), presented in Supplementary Table S3. The data for SO-compromised plants represent the mean for SO Ri 131 and SO Ri 421 mutants.

Examination of nitrogen (N) levels in the top leaves revealed that under normal growth conditions the total N content, detected by elemental analyzer, was lower, while ammonium was significantly higher in the SO Ri mutants as compared to the wild-type plants (Table 2). High ammonium content in the SO Ri mutant leaves could be a result of higher ammonium and nitrate remobilization from the lower plant parts. Indeed, highly elevated levels of ammonium and nitrate were extracted from the stem sap below the top leaves of SO Ri murant as compared to wild-type stem sap (Table 2). The remobilized nitrate was likely reduced in the top leaves to ammonium (Table 2) and amino acids (Figure 5 and Supplementary Table S3). 

**Table 2:** The levels of total, inorganic, organic nitrogen and RNA in top leaves of wild-type (Wt) and SO RNA interference mutants (Ri) plants under normal growth conditions (0 day) or after being exposed to eleven days of extended dark stress (11 day).

**Total, Organic and Inorganic (Nitrate and Ammonium) Nitrogen in the Top Leaves.**									
µmol·g^−1^ Fw		Wt				SO Ri			
		0 day		11 day		0 day		11 day	
Inorganic N	N (nitrate)	65.4 ± 5.6 Ba		98.8 ± 13.2 Ab		44.8 ± 3.7 Bb		133.2 ± 8.4 Aa	
	N (ammonium)	10.3 ± 0.9 Bb		42.3 ± 12.1 Ab		14.8 ± 2.7 Ba		73.4 ± 10.2 Aa	
Organic N		346.9 ± 11.5 Aa		327.6 ± 8.0 Aa		325.2 ± 10.5 Aa		259.7 ± 26.7 Bb	
Total N		422.5 ± 12.5 Ba		468 ± 6.7 Aa		384.7 ± 12.6 Bb		466.3 ± 37.7 Aa	
The levels of nitrate and ammonium in the stem just below the top leaves *									
µmol·mL^−1^ sap		Wt				SO Ri			
		0 day		11 day		0 day		11 day	
Inorganic N	N (nitrate)	0.7 ± 0.07 Bb		10.0 ± 2.6 Ab		1.4 ± 0.4 Ba		15.7 ± 1.7 Aa	
	N (ammonium)	4.3 ± 0.8 Bb		11.4 ± 1.0 Ab		5.0 ± 0.5 Ba		41.4 ± 4.7 Aa	
Total RNA in the top leaves **										
µg·g^−1^ Fw	Wt				SO Ri					
	0 day	11 day	degraded		0 day		11 day		degraded	
RNA	320.0 ± 31.2 Aa	221.9 ± 20.2 Ba	98.1 ± 18.3 b		388.3 ± 18.5 Aa		197.5 ± 24.1 Ba		190.9 ± 15.1 a	

* For nitrate determination in the stem xylem sap, exudate was collected from stem topped 1 cm below the top leaves after applying 6.2 bar for 10 min by a pressure chamber, Arimad 2; ** Degraded RNA was calculated as the RNA level in T0 minus the level in T11. The values denoted with different letters are significantly different according to the Turkey-Kramer HSD test (JMP 8.0 software, [37]; *p* < 0.05, *n* = 3–10). Different lower case letters indicate differences between SO mutant and wild-type plants within the same treatment. Different upper case letters indicate significant difference within the plant genotypes in response to treatment. The data for *SO*-compromised plants represent the mean for SO Ri 131 and SO Ri 421 mutants.

Consistent with this observation, the total amounts of protein-bound and free amino acids were significantly higher (1.13 and 1.49-fold, respectively) in the SO Ri mutants as compared to the wild-type plants when grown under non-stressed conditions (Figure 5, Supplementary Table S3). Importantly, inspection of the S-amino acids in comparison to the other amino acids revealed that for the majority of protein-bound amino acids, the levels were *ca.* 10% higher in the unstressed SO Ri mutants, while the levels of the protein bound Met and Cys were higher by 39% and 84%, respectively. In contrast, the levels of free S-amino acids did not differ much between the unstressed genotypes (Supplementary Table S3).

The extended dark stress resulted in a significant enhancement in the total N in both wild-type and SO mutant plants. Nonetheless, a reduction in the organic N level was noticed in the SO Ri mutants, but not in the wild-type plants. In contrast, a significant increase in the inorganic N content of both plant types was evident after dark stress (Table 2). This is likely a result of the significant enhancement in nitrate and ammonium levels that was much stronger in the SO mutants compared to the wild-type plants. These results are in agreement with the higher degradation levels of total amino acids and RNA in the mutant plants in response to dark stress (Table 2, Figure 5 and Supplementary Table S3). Due to the excessive protein degradation, amino acid deamination and RNA catabolism during the extended dark stress, a greater portion of ammonium was released in the SO Ri mutants, compared to the wild-type plants (Table 2). 

**Figure 6:**
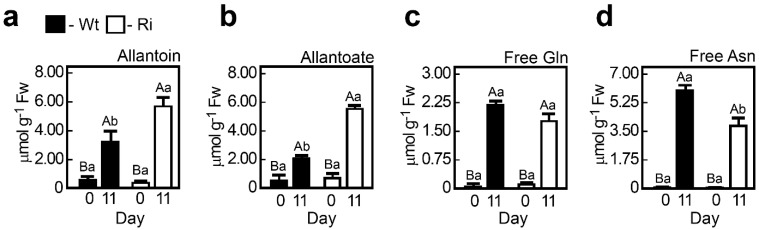
The levels of the ureides (**a**) allantoin and (**b**) allanoate and the amides (**c**) glutamine (Gln) and (**d**) asparagine (Asn) in wild-type (Wt) and SO RNA interference mutants (Ri) plants. Plants were grown under normal growth conditions (0 day) or after being exposed to eleven days of extended dark stress (11 day). The bars are the average values ± SE (*n* = 3–10). The values denoted with different letters are significantly different according to the Turkey-Kramer HSD test (JMP 8.0 software, [37]; *p* < 0.05). Different lower case letters indicate differences between SO mutant and wild-type plants within the same treatment. Different upper case letters indicate a significant difference within the plant genotypes in response to treatment. The data for *SO*-compromised plants represent the mean for SO Ri 131 and SO Ri 421 mutants.

Similarly, the enhancement in the level of the ureides, allantoin and allantoate (Figure 6a,b), as well as in the level of the amides, Gln and Asn (Figure 6c,d), is the result of the dark-induced accelerated catabolism of RNA and protein-bound amino acids, respectively. Impressively, the levels of the amides, Asn and Gln, were significantly enhanced in the wild-type (8.1 µmol (Gln + Asn)·g^−1^ FW) and the mutant leaves (5.9 µmol (Gln + Asn)·g^−1^ FW) in response to the dark stress, representing 81% and 60% of the total level of free non-S-containing amino acids in the wild-type and mutant plants, respectively (see Supplementary Table S3 free amino acids and Figure 6c,d). Ureides and amides are thought to be efficiently remobilized *N*-containing molecules with the advantage of a high N/C ratio (1:1 for the ureides and 1:2 to 1:2.5 for the amides, [12,46]). Interestingly, the increase in the ureide levels was higher in the SO Ri mutants, while the amides content was generally higher in the wild-type plants (Figure 6), likely reflecting the response to a higher level of carbon starvation (stronger need for molecules with 1:1 N:C ratio) in the mutant plants. Accordingly, the higher amount of ammonium in the leaves of the SO Ri mutants supports this notion as it is followed by the dark-induced decrease in sugar content necessary for the ammonium assimilation in plants [47]. Dark treatment forces the plant to use stored forms of sugar and amino acids (AA) as energy. Indeed, the total amount of protein-bound AA was reduced by half in both the wild-type and mutant plants, whereas the free AA were significantly increased to a similar level in both genotypes, yet the total AA was significantly decreased more in the SO Ri mutants as compared to the dark-stressed wild-type plants (Figure 5 and Supplementary Table S3). 

### 2.2. Discussion

#### 2.2.1. The Absence of SO Activity Alters Sulfur Metabolism in the Tomato Plants Grown under Normal Growth Conditions

Our results indicate that the SO mutation significantly affects broad aspects of metabolism, revealing hitherto unsuspected effects of this lesion. Furthermore, under induced catabolic processes, triggered by extended dark stress, further differences are noted. 

**Figure 7:**
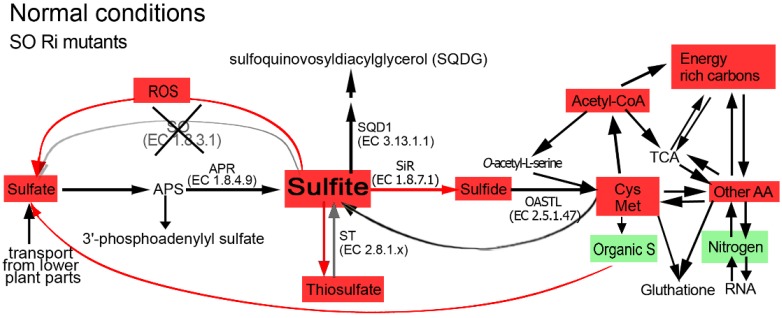
Schematic illustration describing the impact of sulfite oxidase (SO) impairment on the sulfur, carbon and nitrogen metabolism in tomato plants grown under normal growth conditions. Abbreviations are: adenosine-5′-phosphosulfate (APS), APS reductase (APR), sulfite reductase (SiR) *O*-acetyl-serine-thiol-lyase (OAS-TL), UDP-sulfoquinovose synthase (SQD1), sulfite oxidase (SO), sulfurtransferases (ST). The Cys Met box indicates the sum of total Cys and Met. The other AA indicates the total detected amino acids other than Cys and Met. Energy rich carbon indicates fatty acids, sucrose, fructose, sorbitol and myoinosytol. TCA indicates pyruvate and the following TCA cycle metabolites: citrate, α-ketoglutarate, succinate, fumarate and malate. Nitrogen indicates the total N as in Table 2. Organic S indicates total organic S as in Supplementary Table S2. ROS indicates the reactive oxygen species as in Supplementary Figure S3. Red and green colored boxes indicate an enhancement or a reduction in metabolite content, respectively, as compared to those in the unstressed top leaves of the wild-type plants. The non-colored metabolites indicate similar levels as compared to the unstressed top leaves of the wild-type plants. The red arrow indicates enhanced SiR activity as compared to wild-type. The grey arrow indicates decreased sulfite oxidation activity due to SO impairment.

The absence of tissue damage or visible modified phenotypes in the SO mutant plants grown under normal growth conditions indicates sufficient sulfite detoxification [14,19,21,23,24]. However, the SO mutation alters C, N and S metabolism in plants grown under normal unstressed growth conditions (Figure 1, Figure 2, Figure 3, Figure 4, Figure 5, Figure 6 and Figure 7, Table 2, Supplementary Tables S2 and S3). The SO-impaired plants exhibit significantly higher levels of sulfide, total Cys and Met as compared to the wild-type plants (Figure 1). This is attributed to the channeling of excess sulfite towards its reduction by enhanced SiR activity in the presence of high NADPH level (see Figure 3a, Supplementary Supplementary Figure S3 and S5e in [14], respectively). The higher acetyl-CoA and Serine (Ser) contents in the mutant plants supports the elevated Cys and Met biosynthesis (Figure 1, Figure 5 and Figure 7, Supplementary Tables S2 and S3). Taken together, our results indicate that the impairment in tomato SO activity leads to an imbalance in sulfite homeostasis which is compensated by a metabolic shift towards a futile sulfite reduction pathway, leading to elevated levels of reduced sulfate-type compounds.

A similar total sulfur content was evident in both genotypes (Figure 1, Supplementary Table S2) yet the distribution differed. Higher sulfide, Co-A, acetyl Co-A, total Cys and Met levels were evident in the mutants, and similar levels of SQDG and total glutathione were noted compared to the wild type. Glutathione functions as a major reservoir for reduced sulfur, however, due to its intrinsic involvement in the cellular redox state its level is tightly controlled. Significantly lower total organic sulfur levels were found in the mutant as compared to the wild-type leaves (Figure 1 and Figure 7, Supplementary Table S2) indicating less efficient sulfur assimilation in plants with inactive SO. Considering also the higher inorganic oxidized sulfur levels (thiosulfate, sulfate and sulfite) in the mutant plants, the results indicate the presence of a residual footprint of anabolism (metabolic shift towards S-AA) and catabolism (degradation of organic S metabolites) in the SO-impaired plants. 

The level of sulfate was significantly higher in the SO mutant top leaves as compared to the top leaves in the unstressed wild-type plants (Figure 1 and Figure 7, here and Supplementary Table S1 in [14]). What can be the cause of the higher sulfate in mutant plants with impaired sulfite oxidase activity? Considering the similar total sulfur levels detected in both wild-type and SO mutant top leaves (Figure 1, Supplementary Table S2 here and Supplementary Table S1 in [14]), this can be attributed to the following: (i) the high ROS levels in mutant leaves (Supplementary Figure S3) that could participate in the oxidation of the excess sulfite to sulfate [25] and/or (ii) the product of organic-S metabolites enhanced degradation in the SO-impaired plants (Figure 7).

#### 2.2.2. Growth Is Impaired in the SO Mutant Plants Due to a Futile Sulfite Reduction Pathway and the Generation of High Levels of Cellular Macromolecules and Energy Resource Metabolites

Although no obvious tissue damage was noticed in the mutant plants grown under normal growth conditions, a reduction of 16.5% of total plant biomass as compared to the wild-type plants was detected (Supplementary Figure S1a), while the expression level of stress marker genes was similar or lower as compared to wild type (Supplemental Figure S1b), indicating that mutant plants were not stressed. This can be attributed to the following: (i) energy rich metabolites (e.g., ATP and reduced glutathione) normally employed for metabolism and growth [48] are consumed due to the futile sulfite reduction mentioned above and (ii) the generation of higher levels of cellular macromolecules in the mutants as compared to the wild-type plants. Such macromolecules include total RNA (Table 2) and total protein, as can be seen from the higher total content of protein-bound amino acids in the unstressed mutant leaves (Figure 5, Supplementary Table S3). In this context, the significantly higher ROS levels in the unstressed mutant plants as compared to the wild-type plants (3-fold enhancement (Supplementary Figure S3)) are likely indicative of the enhanced expression of the mitochondrial electron transport chain needed for the futile generation of the energy rich metabolites in the mutant plants, that generates ROS as a by-product [49]. The lower efficiency of the mutant metabolism was further demonstrated by the higher content of energy resource metabolites such as carbohydrates (sucrose and fructose), as well as alcohols, sorbitol and myoinositol as compared to wild-type plant tissues (Figure 2). A high sugar content may suggest a high rate of amino acid and fatty acid biosynthesis [50,51], as the enhanced resulting products are shown here (Supplementary Table S3, Figure 4a).

#### 2.2.3. Enhancement in Total S-amino Acids Alters Total Amino Acids Level in SO Mutants

Notably, the enhancement in total S-amino acid levels (Table 1) significantly altered amino acid homeostasis, resulting in an increased content of total protein-bound and free amino acids in the tomato SO-impaired plants as compared to the unstressed wild-type plants (Figure 5 and Figure 7). Hence, Cys and/or Met could be involved in the control of non-S-amino acid levels in the unstressed plants. Similarly, the increase of Met in modified tobacco and soybean seeds resulted in an increase of most other amino acids in the transgenic seeds [52]. In contrast, the increase in Cys and Met in modified alfalfa leaves led to a selective change in aspartate and lysine levels, but no significant difference was noted in total amino acids [53]. These results indicate that there are complex regulatory steps to allocate carbon and nitrogen sources that respond to the altered S-amino acid content that may differ between species.

#### 2.2.4. Phospholipid Content Is Significantly Enhanced in the SO Mutant Plants

A major difference in the fatty acid content under control unstressed conditions is evident by the significantly elevated phospholipid levels in the mutant plants (Figure 4f). Phospholipids have emerged as important secondary messengers [54] that regulate plant growth, development and cellular responses to environmental change or stress [55,56,57,58,59]. The significant increase in phospholipid content in the SO-impaired plants was mainly due to the 2.4- and 1.3-fold increase in PE and PG, respectively, as compared to the wild-type plants (Figure 4f–i). PE is a major bilayer lipid and is essential for the proper nutrient recycling and timely onset of senescence, as conjugation of PE and the ubiquitin-like protein ATG8 produces a complex that is essential for autophagosome formation [60]. The higher level of PE in the SO Ri mutants under normal growth conditions may be correlated with elevated ATG8 as compared to the wild-type (Supplementary Figure S4). Such regulation of ATG8 by elevated PE is proposed to ensure the autophagosome formation [60], which could be essential for enhanced nutrient recycling in the mutant plants. 

#### 2.2.5. The Absence of SO Confers High Mortality Rate and Reduced Biomass Accumulation in Tomato Plants Exposed to Extended Dark Stress

To a certain extent, prolonged dark treatment mimics natural senescence [12,14,61]. However, if a plant is impaired in enzymes that have pivotal roles in plant catabolism one can expect higher mortality rates among the mutants during dark stress than those observed in the wild-type plants [12,14,61]. In accordance with this notion, extended dark stress resulted in a significantly lower biomass accumulation in the surviving mutant plants (Supplementary Figure S1) and a higher mortality rate among the SO-impaired plants as compared to the wild-type plants (60% to 70% in the mutants *vs.*10% in the wild-type plants (see [14]). It has been shown that SO is important for plant survival during the prolonged dark period due to the ability to directly detoxify sulfite originated from degraded biomolecules [14].

#### 2.2.6. Extended Dark Stress Leads to Accelerated Catabolism of S Metabolites and Differently Alters S and non-S amino Acids Levels and Their Ratio in SO Mutants as Compared to Wild-Type Tomato Plants

**Figure 8:**
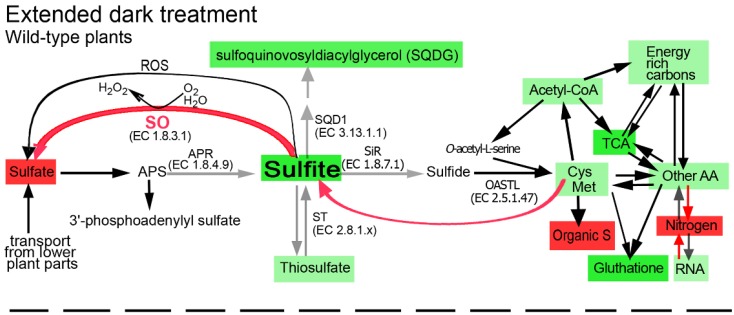

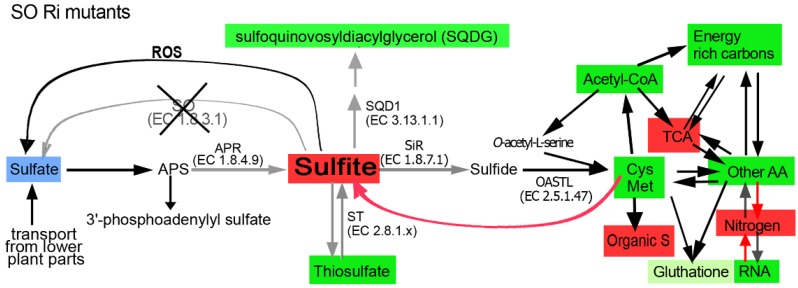
Schematic illustration describing the impact of sulfite oxidase (SO) impairment on the sulfur, carbon and nitrogen metabolism in the top leaves of tomato plants grown for 11 days in dark. Abbreviations are: adenosine-5′-phosphosulfate (APS), APS reductase (APR), sulfite reductase (SiR) *O*-acetyl-serine-thiol-lyase (OAS-TL), UDP-sulfoquinovose synthase (SQD1), sulfite oxidase (SO), sulfurtransferases (ST). The Cys Met box indicates the sum of total Cys and Met. The other AA indicates the total detected amino acids other than Cys and Met. Energy rich carbon indicates fatty acids, sucrose, fructose, sorbitol and myoinosytol. TCA indicates pyruvate and the following TCA cycle metabolites: citrate, α-ketoglutarate, succinate, fumarate and malate. Nitrogen indicates the total N as in Table 2. Organic S indicates total organic S as in Supplementary Table S2. ROS indicates the reactive oxygen species as in Supplementary Figure S3. The blue box indicates that the sulfate level was not affected by the stress but was significantly lower in the mutant as compared with the stressed wild-type top leaves [14]. Red or green colors indicate the enhancement or reduction, respectively, in metabolites, as compared to the contents in the unstressed top leaves of the wild-type plants (light green indicates less reduction compared to the darker green). The red arrow from the Cys Met box indicates the direction of sulfite, the degradation product of these amino acids [14]. Red or grey arrows indicate the SO activity enhancement or decrease, respectively. The lack of colored metabolites or colored arrows indicates similar levels as compared to the unstressed top leaves of the wild-type plants.

The mutant plants exposed to prolonged dark stress exhibited enhanced degradation of S-amino acids and their building blocks (Figure 1, Supplementary Table S2). This was evident with acetyl-CoA and serine, needed for the biosynthesis of O-acetyl serine, as well as with sulfide, both being substrates for cysteine biosynthesis (Supplementary Tables S2 and S3, Figure 8). This indicates that the content of S-amino acids was decreased not only by the enhanced catabolic processes but also by suppression of their *de novo* biosynthesis. The latter could be noticed by the residual activities of SiR and APR, albeit considerable, yet significantly decreased in response to the prolonged dark stress [14].

Impressively, the dark-induced degradation of S-amino acids in the wild-type plants resulted in a similar rate of non-S-amino acid degradation and thus S-amino acids to non-S-amino acids ratios in stressed and unstressed growth conditions were identical (Supplementary Table S5). In contrast, in the absence of SO activity, a higher dark-induced degradation rate of S-amino acids was noted as compared to non-S-amino acids (61% *vs.* 37%, respectively). This results in a significantly lower ratio of S-amino acids to non-S-amino acids as compared to that in the wild-type plants (1.7% *vs.* 2.1%, respectively, (Supplementary Tables S2 and S5 and Figure 8)). These results indicate that the normal SO activity is important for the control of Cys and/or Met biosynthesis not only in normal growth conditions, but also during carbon starvation induced senescence. Considering also the significant enhancement in the ratio of S to non-S-amino amino acids in SO Ri as compared to the unstressed wild-type plants (2.8% *vs.* 2.1%, respectively (Supplementary Table S5)), these results indicate that in the absence of SO activity the biosynthesis or degradation of the amino acids is specific for the S and non-S amino acids, in a C availability depended manner.

#### 2.2.7. The Accumulation of Toxic Sulfite and the Carbon Starvation Lead to a Higher Acceleration of Catabolism in the Dark-Stressed Mutant as Compared to the Tomato Wild-Type Plants

The higher degradation rate of S-amino acids in the dark-treated mutants resulted in the accumulation of toxic levels of sulfite as opposed to the dark-stressed wild-type plants [14]. Sulfite, as a strong nucleophile, can deleteriously react with a wide variety of cellular components, such as plant tissue, organelles, macromolecules and metabolites, to form sulfated compounds, so-called other-S-compounds (calculated as the difference between known S metabolites to the total content of sulfur) [14,19,62,63,64,65]. Indeed the other-S-compounds were elevated in the dark-stressed SO mutants (Figure 1, Supplementary Table S2). Yet, the prolonged dark stress is characterized by massive degradation of biomolecules [12,14,61] not only in the SO mutants but in the wild-type plants as well (Figure 1, Figure 2, Figure 4, Figure 5, Figure 8 and Table 2). This degradation is attributed mainly to the dark-induced low sugar availability, resulting in fatty acid oxidation through peroxisomal β-oxidation, as well as cellular proteins and entire organelle degradation by autophagy to employ the resultant amino acids as an energy source [66,67]. With this *caveat* in mind, the more prominent decrease in energy source metabolites such as NADPH (see Figure 5e in [14]), total fatty acids and amino acids in the mutant plants (Figure 2, Figure 4, Figure 5 and Figure 8) is probably the result of the higher essentiality of detoxification processes. These may include, in addition to sulfite, the toxic chlorophyll breakdown intermediates [68,69,70,71,72], ammonium [73], ROS [74] and toxic lipid peroxidation products such as malondialdehyde [15] and others. The reduced biosynthesis of fatty acids in the SO mutants, as indicated by the accumulation of pyruvate (Figure 3a), the decreased levels of acetyl-CoA (Figure 1, Supplementary Table S2), and the increase in the TCA intermediates (Figure 3 and Figure 8), means a failure to adjust to cataplerotic-type respiration for essential carbon backbones (see below) in the dark-stressed mutant plants, further contributing to the energy shortage. These results indicate that the absence of SO activity in the mutant plants results in the accumulation of toxic sulfite that leads to a higher acceleration of the catabolism as compared to the wild-type plants by deleteriously reacting with and degrading a wide variety of cellular components, causing additional energy shortage by the generation of toxic degrading products essential to be detoxified. The inability of the mutants to cope with the cascade of toxic metabolites leads to premature senescence and higher mortality rate in the mutants as compared to the wild-type plants [14].

#### 2.2.8. Extended Dark Stress Leads to Enhanced Degradation of Organic Nitrogen, Elevated Ammonium and Preference for Lower C/N Ratio Metabolites in the SO Mutants as Compared with the Wild-Type Plants

The higher ammonium level in the leaves of the dark-stressed SO mutants (Table 2) is most likely the product of the amino acid degradation (Supplementary Table S3), known to be remobilized during leaf senescence [75], rather than being a significant sink for amino acid *de-novo* synthesis [2,76]. An additional source of ammonium in the SO mutants is the enhanced nucleic acid catabolism [77] as evident from the significantly higher total RNA degradation and the enhancement of the ureides (allantoine and allantoate) as compared to the wild-type plants (Table 2 and Figure 6a,b). The massive degradation of total RNA detected here is indicative of the deficit in the availability of resources to be invested in the machinery that turns metabolites into ribosomal RNA, resulting in the degradation of the RNA, as well as the degradation of the ribosomal proteins especially in young growing leaves [48]. The relatively high ammonium level is most likely an additive stress [78,79], subsequent to that of the toxic sulfite level, which causes further senescence progression in the dark-stressed SO-impaired plants [14].

Glutamine synthetase (GS) is a key enzyme, because it catalyses the adenosine triphosphate (ATP)-dependent fixation of ammonium to glutamate to form glutamine. The cytosolic isoform of GS, GS1, is particularly important for assimilating ammonium for the primary nitrogen assimilation and recycling. Glutamine is the substrate for glutamate synthase (GOGAT), where, by employing NADH, the cytosolic form of the enzyme catalyses the conversion of glutamine and 2-oxoglutarate to two molecules of glutamate that can be employed for further ammonium assimilation and/or incorporation into other amino acids [80]. The stronger dark-induced up-regulation of the cytosolic GS1 and NADH-GOGAT transcripts in the mutant leaves is indicative of the higher energy cost (ATP and NADH) for detoxifying each ammonium molecule, as compared to the wild-type GS-GOGAT cycle (Supplementary Figure S5a,b).

Similarly, it is possible that the enhancement of glutamate dehydrogenase1 (GDH1) and GDH2 in SO mutants, as compared to the wild-type transcripts (Supplementary Figure S5c,d), is indicative of a higher consumption rate of NADH to detoxify ammonium, employing α-ketoglutarate to synthesize glutamate [80]. Yet, the mitochondrial GDHs have been described as mainly playing a role in the generation of ammonium derived from the catabolism of amino acids [80]. Considering the higher degradation rate of total amino acids, specifically total glutamate, in the mutant leaves (Supplementary Table S3), it is possible that the high GDH1 and GDH2 levels indicate the existence of *de-novo* biosynthesis of NADH in an effort to replenish the more exhausted energy pool in the mutant plants during the extended darkness, releasing toxic ammonium resulting from the degradation of glutamate as a substrate.

Ureides, the product of purine catabolism, serve the dual function of reactive oxygen scavengers as well as maintaining a favorable C/N ratio (1:1) [12], especially when carbon assimilates are in shortage, as is expected during extended dark stress (Figure 2a). Interestingly, while the N-rich amides, glutamine and asparagine, with the C/N ratio of 2.5 and 2, respectively, were generally more increased in the wild-type plants than in the dark-stressed mutants, the increase in ureide levels during the extended dark stress was larger in the mutants than in the wild-type plants. These results are indicative of the more severe shortage of carbon assimilates in the mutant plants as compared to the dark-stressed wild-type plants (Figure 2a). This notion is supported by the stronger dark-induced enhancement of *xanthine dehydrogenase* *1* (*XDH1*) transcript level in the mutant leaves as compared to the wild-type leaves (Supplementary Figure S5e), indicating the possible enhancement of NADH biosynthesis by XDH1 activity [81,82], at the same time also encorporating the committed biosynthetic step toward the generation of higher levels of ureides [12]. Considering the significantly higher dark-induced mortality rate in the mutants as compared to the wild-type plants [14], the enhanced efforts to generate NADH as indicated by the enhancement of *XDH1*, *GDH1* and *GDH2* transcripts, were not enough to overcome the bigger shortage of energy rich metabolites in the dark-stressed mutant plants. Additionally, the high ureide levels in the mutants likely exceeded their optimum level for delaying chlorophyll degradation [12] and thus did not contribute to the survival of the mutant plants.

#### 2.2.9. TCA Intermediates in Dark-Stressed Tomato Plants

The dramatic increase in the levels of TCA intermediates represents a major and significant metabolic change in the dark-stressed mutant plants compared to the similarly treated wild-type plants, that exhibit a general decrease in TCA intermediates levels (Figure 3 and Figure 8). Such an increase may represent failure to adjust to cataplerotic-type respiration, *i.e.*, the prolonged dark stress induces the need for carbon backbones. In this context, the significantly stronger dark-induced ROS decrease in the mutants as compared to the wild-type leaves (Supplementary Figure S3) is likely indicative of the reduced expression of the mitochondrial electron transport chain that generates ROS as a by-product [49]. 

The dark-induced accumulation of pyruvate and TCA cycle intermediates in the SO-impaired plants (Figure 3) suggests a diminished ability to use carbon skeletons from the TCA cycle for the down-stream organic compounds biosynthesis. What can trigger this failure in the SO-deficient plants? Trivially, the direct sulfite toxicity may slow down or prevent the use of TCA intermediates, depending on the level of the accumulated sulfite. However, the concomitant rise in the TCA intermediates may suggest additional reasons. For example, amino acid turnover may be less effective in feeding the dark-starved tissue in the mutants. As an example, lysine turnover, an important source of supply for replenishing TCA intermediates [83], is especially reduced in the mutants, reflecting the futile attempt to enhance cataplerotic-type respiration. Moreover, a higher lysine degradation rate can severely compromise the alternative mitochondrial complex, supporting respiration during carbon starvation, resulting in TCA intermediates accumulation [28,49].

#### 2.2.10. The Effect of Dark-Induced Senescence on the Galactolipids and Phospholipids in Tomato Plants

During leaf senescence, the degradation of galactolipids precedes the degradation of phospholipids [84,85,86,87,88,89]. Indeed, the galactolipids were decreased in both genotypes in response to dark stress, with a stronger decrease noted in the mutant plants (Figure 4b). Yet, phospholipids were elevated in the wild-type plants (Figure 4f), likely indicating that these plants were only in the early stages of senescence [88,89]. In contrast, the SO mutant plants exhibited severely decreased phospholipid levels (Figure 4f), consistent with a late senescence stage [41,89]. A major difference in the phospholipid content under stressed conditions lies in the significant decrease of PE in the mutant plants (Figure 4f–i) that could be related to senescence processes accelerating phospholipid degradation [90,91,92,93]. The decrease in the phospholipid content in the mutant leaves paralleled a reduction in the ATG8 protein content (Supplementary Figure S4), suggesting a disruption of the autophagosome function and reduced remobilization capacity of nutritional metabolites. These processes result in accelerated senescence and higher mortality rates of the mutant plants [60,90,91,92,93].

### 2.3. Conclusions

Overall our results indicate that SO activity is essential to maintain optimal carbon, nitrogen and sulfur metabolism in stressed and unstressed tomato plants. Under normal growth conditions, SO activity is essential to ensure normal S metabolism in plants. The absence of SO implies an imbalanced sulfite homeostasis and a metabolic shift towards a futile sulfite reduction pathway, leading to the enhancement in S-amino acids (Figure 7). When plants are exposed to extended dark stress, the enhanced catabolism and inability of the mutant plants to detoxify the accumulated sulfite resulting from the degradation of S-containing metabolites, leads to a higher acceleration of catabolism rates in the SO mutant plants that include higher degradation rates of carbohydrates, amino acids, total RNA and lipids as compared to the wild-type plants (Figure 8). The results also indicate that in the absence of SO activity the biosynthesis or degradation of the amino acids is specific for the S and non-S amino acids, in a C availability dependent manner.

## 3. Experimental Section

### 3.1. Plant Growth Conditions and Experimental Setup

Tomato plants (*Lycopersicon esculentum/Solanum lycopersicum* Mill. *cv. Rheinlands Ruhm*), wild-type and *SO* RNA interference (*SO Ri*) mutants [14] that harbor indeterminate growth pattern were grown in pots filled with a peat and vermiculite (4:1 v/v) mixture containing slow-release high-N Multicote 4 with micro-elements (0.3% w/w; Haifa Chemicals Ltd., Haifa, Israel, [94]) in a growth room under 16-h light/8-h dark, 22 °C, 75%–85% relative humidity, and with 100 μE·m^−2^·s^−1^, as previously described [14]. For dark treatment, 6-week-old tomato plants were transferred to a dark room. The samples were collected before the dark treatment and after 11 day of dark conditions as a mix of top leaves (fifth and six from the bottom) taken from five wild-type as well as from each of the two SO RNA interference plants (SO Ri 131 and SO Ri 421) and subjected to the analysis described previously [14] and here.

### 3.2. Extraction and Determination of Polar Lipids

Pre-weighted leaf samples were grounded in liquid nitrogen and were treated with hot isopropanol at 80 °C for 15 min followed by lipid extraction as described in Bligh and Dyer [95]. Total lipid extract was separated into neutral and polar lipid fractions on silica Bond-Elute cartridges (Agilent, Santa Clara, CA, USA, [96]) by sequential elution with chloroform: methanol (99:1, v/v) and methanol, respectively. The polar lipid fraction was dissolved in chloroform prior to the separation into individual lipid classes by two-dimensional TLC (2D TLC). The 2D TLC was performed in a solvent system of chloroform:methanol:water (65:25:4, v/v/v) for the first direction and of chloroform:methanol:isolpropylamine:conc. ammonia (65:35:0.5:5, v/v/v/v) for the second direction. For qualitative assessment, glycoglycerolipids were visualized by spraying with 2.4% α-naptol solution in 80% ethanol with 10% sulfuric acid followed by heating at 120 °C for 10 min. For individual polar lipids quantification, lipid spots on TLC plates were visualized with 0.05% 8-anilino-4-naphthosulphonic acid in methanol (w/v) and UV light exposure, and then scraped from the plates and transmethylated for fatty acid analysis. Total fatty acids as well as individual lipid spots were transmethylated with 2% sulfuric acid in absolute methanol and FA methyl esters (FAME) were extracted to hexane as previously described by us [42,97]. The FA profile and contents of total and individual lipid classes were determined as FAME by GC-FID on Trace GC Ultra (Thermo Scientific, Waltham, MA, USA, [98]) as previously described [42]. 

### 3.3. Determination of Total C, Total S, Inorganic and Total N Content

In order to quantify the total nitrogen and carbon, about 4.6 mg of the powdered leaves were placed in tin containers in elemental analyzer (EA1108 CHNS/O; Fisons Instruments, Milan, Italy) and the amounts of total N and C in each sample were quantified according to a standard calibration curve prepared with cystine. Total S content was determined as described before [99]. 

Inorganic nitrate was determined by an ion exchange chromatography system (DX 600; Dionex, Waltham, MA, USA, [100]) using an IonPac column (AS 4ASC; Dionex [100]) for separation and an electrochemical conductivity detector (ED 50; Dionex, [100]) combined with an upstream-inserted micromembrane suppressor (ASRS-Ultra II 4 min; Dionex, [100]).

Exudate sap was collected from plants topped 1 cm below the fifth leaf. The entire root system of the tomato plants was washed with distilled water, blotted dry with filter paper, and transferred into an Arimad 2 pressure chamber (MRC Ltd., London, UK, [101]). Thereafter, the pressure was gradually increased to cause exudation. The sap (approximately 200 μL) was collected [102,103,104] under permanent pressure of 6.2 bar during 10 min and then frozen in liquid nitrogen and kept in—80 °C before use for metabolites determination.

Ammonium from leaf samples was extracted in double distilled water (1:200 (w/v) on ice and then determined with an ammonium test kit (114657, Merck, Billerica, MA, USA, [105]) as previously described by us [106], followed by measurement at 700 nm. Ammonium was quantified according to a standard calibration curve prepared with NH_4_Cl.

### 3.4. Quantification of Total and Free Amino Acids, GSH, CoA and Acetyl-CoA

For protein-bound amino acid determination, total proteins were extracted from 100 mg leaves using a standard TCA precipitation protocol [107], followed by washing the sodium dodecyl sulfate (SDS) three times with 100% methanol and 80% acetone. Extracted proteins were hydrolyzed in constant boiling HCl vapors at 110 °C, for 22 h under nitrogen [108]. Total amino acids were determined by the AccQ·Tag method (Waters Corporation, Milford, MA, USA, [109]) using a Waters Alliance 2695 high performance liquid chromatography (HPLC) Instrument. Quantification was performed using Waters 2475 Multi 22 Wavelength Fluorescence detector as previously described [14,110,111]. Free amino acids were extracted and detected according to [108,112], as previously described [14]. Reduced and oxidized forms of glutathione were also determined according to Griffith [113] with similar results to those detected by HPLC. To determine acetyl-CoA and CoA, samples were deproteinized using 10KD molecular weight cut-off spin column (BioVision, Milpitas, CA, USA, [114]). Levels of Acetyl-CoA and CoA were evaluated according to the manufacturer’s instructions (Coenzyme A (CoA) using a Colorimetric/Fluorometric Assay Kit, BioVision [114]) at 570 nm *versus* the standard curve based on CoA.

### 3.5. Extraction and Determination of Sugars, Alcohols, Pyruvate and TCA Metabolites

Metabolite extraction was performed as previously described [115,116]. Leaf samples (100 mg) were homogenized using a precooled mortar and pestle with liquid nitrogen and extracted in 100% methanol. Ribitol (0.2 mg/mL) was added as an internal quantitative standard for the polar phase. Metabolites from the supernatant fraction were further extracted with CHCl3:H_2_O (1:2). The upper polar phase was derivatized and analysed using an established GC-MS based method, as previously described [115,116]. In brief, 40 μL of freshly prepared methoxyaminhydrochloride in pyridine (20 mg·mL^−1^) was added to each sample followed by continuous shaking at 37 °C for 2 h. Seventy μL of *N*-methyl-*N*-trifluoroacetamide and 7 µL of alkane mixture pre-warmed to 70 °C were added to the samples, which were then agitated for 30 min at 37 °C followed by transfer to autosampler vials (Thermo Scientific, [98]). Separation was carried out on a Thermo Scientific DSQ II GC/MS using a FactorFour Capillary VF-5ms column. Mass spectral searching utilized the National Institute of Standards and Technology (NIST, Gaithersburg, MD, USA) algorithm incorporated in the Xcalibur^®^ data system (version 2.0.7) against RI libraries downloadable from the Max-Planck Institute for Plant Physiology in Golm, Germany [117]. The level of metabolites was calculated by normalizing the intensity of the peak of each metabolite to the ribitol standard and tissue fresh weight. 

### 3.6. ROS Production

H_2_O_2_ was detected with 0.85 mM 4-aminoantipyrine, 3.4 mM 3,5-dichloro-2-hydroxobenzene sulfonate, 4.5 U mL^−1^ HRP in 200 μL of 50 mM phosphate buffer (pH 7.5) in the presence of 2 mM tungstic acid and 1 mM DPI as previously described [12,24,82]. Hydrogen peroxide concentrations were calculated *versus* H_2_O_2_ standard curve. Superoxide was assayed spectrophotometrically by measuring the oxidation of epinephrine to adrenochrome at 480 nm, using CuZn-SOD as a blank in the presence of 2 mM tungstic acid and 1mM DPI as previously described [82,118].

### 3.7. Preparation and Quantification of RNA

Total RNA was extracted using Aurum total RNA mini kit (Bio-Rad, Hercules, CA, USA, [119]) as previously described [14,19]. RNA concentration was determined using a NanoDrop ND-1000 spectrophotometer (Nanodrop ThermoScientific, Wilmington, DE, USA, [120]).

### 3.8. Quantitative Real-Time Reverse Transcription

For quantitative analysis of transcripts expression, RT reaction with oligo(dT)/random hexamer primers and quantitative RT-PCR reactions employing specific primers (see in Supplementary Table S4) were performed as described before [14,19]. Reactions normalized with *ACTIN Tom41* (U60480), *elongation factor 1-α* (SGN-U196120) and *TFIID* (SGN-U329249) as housekeeping genes revealed similar results, allowing us to present results based on the *TFIID*.

### 3.9. Protein Extraction and Immunoblot for ATG8 Proteins

For immunoblot analysis of ATG8, proteins from leaf samples were extracted, and equal amount of proteins (Bradford assay [121]) were fractionated in 15% SDS-PAGE and blotted to polyvinylidene difluoride membranes (Immun-Blot membranes, Bio-Rad, [119]) [14]. Blotted proteins were subjected to immunodetection with specific antisera raised against polyclonal anti-ATG8 antibody (ab98830; Abcam, Cambridge, UK, [122]) in 1:1000 ratio followed by binding with 5000-fold secondary antibodies diluted in PBS (anti-rabbit IgG; Sigma-Aldrich, St. Louis, MO, USA, [123]). Protein bands were visualized by staining with ECL system SuperSignal Western Blotting System (Pierce-Life Technologies, Carlsbad, CA, USA, [124]) and quantified by NIH Image software (version 1.6).

### 3.10. Multivariate and Statistical Analysis

The data for the *SO Ri* plants represent the mean for *SO Ri* 131 and *SO Ri* 421 lines, three to four independent biological replications per line, where each replication is a bulk of five independent plants. The data for the WT plants represent mean obtained from four to eight independent biological replications, where each replication is a bulk of five independent plants. Each treatment was evaluated using ANOVA (JMP 8.0 software, [37]). Metabolite measurements were done on three to ten independent experiments.

Principal component analysis (PCA) was performed on the data sets obtained from different analytical platforms (GC-MS, HPLC, Ion exchange chromatography, elemental analyzer, AccQTag system, GC Ultra, colorimetric detection as described above) with the software package tMEV [125,126]. Prior to the analysis, data were integrated by median normalization of the entire sample set for each parameter [127]. Considering the fact that a different number of replicates was used for different types of analysis (*n* = 3 to 10, depending on the type and platform used for the metabolites detection) three representative replicates of each sample were selected according to a simple random sampling model [128] in freely distributed R-project (version 2.15.1).

## Supplementary Files

Supplementary File 1
