# Supplementary material for: Sulfite Oxidase Activity Is Essential for Normal Sulfur, Nitrogen and Carbon Metabolism in Tomato Leaves
Source: Plants (Basel). 2015 Aug 14;4(3):573–605. doi: 10.3390/plants4030573 (PMC4844397; doi:10.3390/plants4030573)
Supplement: Supplementary File 1 [file plants-04-00573-s001.zip › plants-89629-supplementary-final/plants-89629-supplementary-layout.pdf]

Supplementary Materials

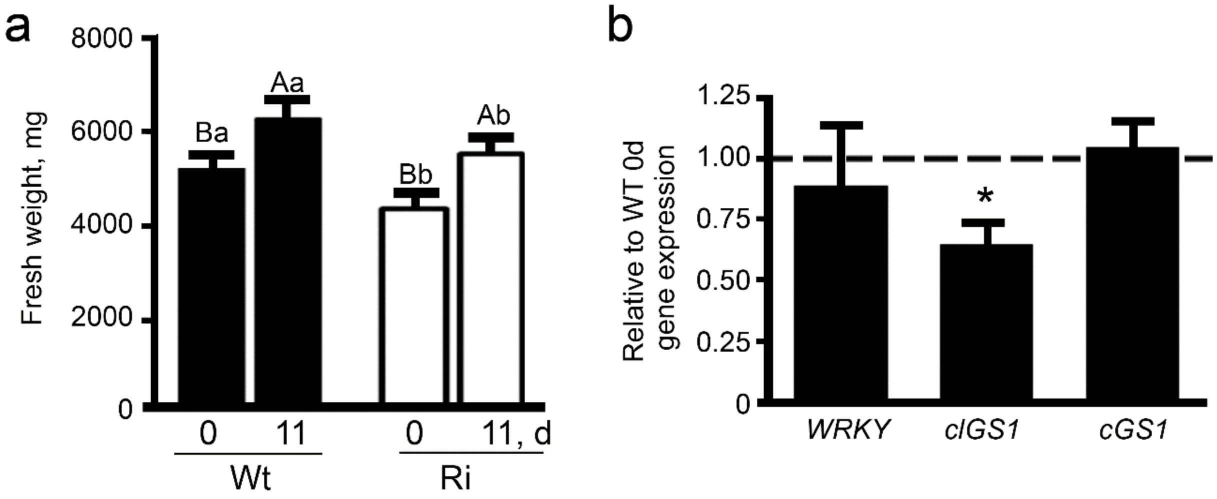

Supplementary Figure S1. (**a**) Total biomass accumulation (roots and upper plant part) in wild-type (Wt) and SO RNA interference mutants (Ri) plants grown under normal growth conditions (0 day) and after being exposed to eleven days extended dark stress (11 day). The bars are the average values ± SE (*n* = 10 individual experiments). The values denoted with different letters are significantly different according to the Turkey-Kramer HSD test (JMP 8.0 software, http://www.jmp.com/; $p < 0.05$). Different lower case letters indicate differences between SO mutant and wild-type plants within the same treatment. Different upper case letters indicate significant difference within the plant genotypes in response to treatment; (**b**) Relative to wild type (T0) expression of senescence marker genes *WRKY transcription factor 2d-1* (*WRKY*), *chloroplast glutamine synthetase1* (*ClGS1*) and *cytosolic glutamine synthetase (cGS1)* in SO mutants. The bars are the average values ± SE (*n* = 3 individual experiments). Significance of the results (*) was calculated by Student's *t*-test (JMP 8.0 software, http://www.jmp.com/; $p < 0.05$). The data for SO-compromised plants represent the mean for SO Ri 131 and SO Ri 421 mutants.

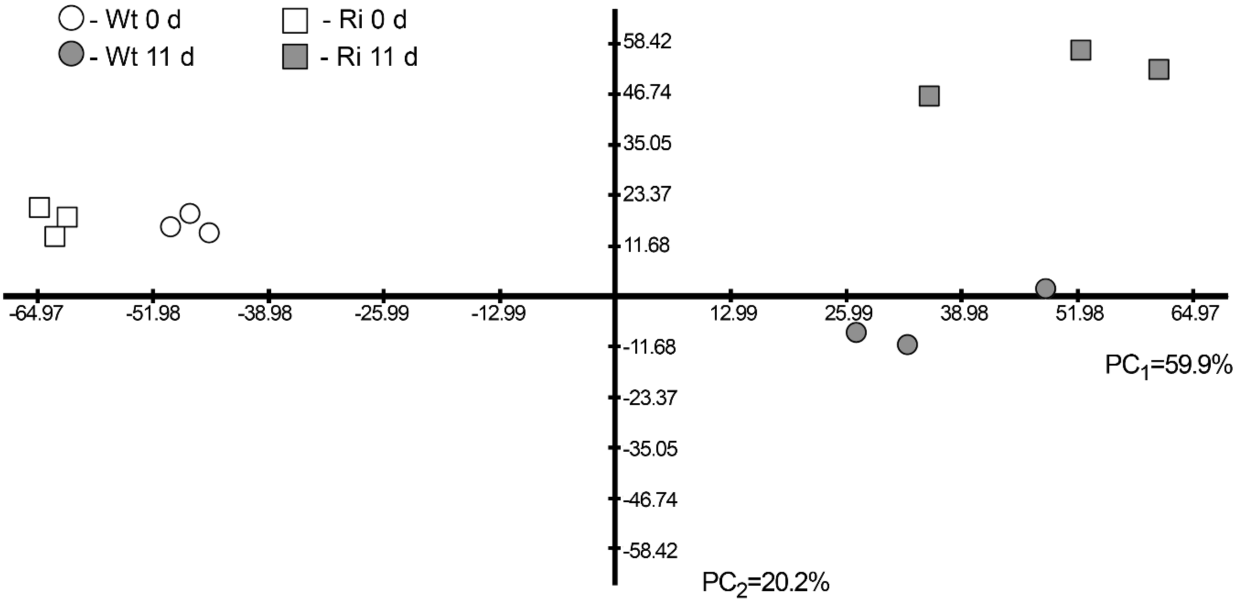

Supplementary Figure S2. Principal component analysis of C, N and S metabolites detected in wild-type and SO RNA interference (Ri) tomato mutants during normal growth conditions (0 d) and dark stress (11 day, see legend on the plot). First principal component (PC_1) and second principal component (PC_2) are plotted on the axes. The variance explained by each component is indicated on the plot. The three representative replicates of each sample were selected from 3 to 8 independent replicates (see Experimental Section) according to a simple random sampling model (http://www.gobookee.net/practice-of-statistics-daniel-yates) in freely distributed R-project (version 2.15.1), integrated by median normalization of the entire sample set for each parameter. The PCA plot was built in software package tMEV (http://www.tm4.org/). The data for the *SO*-compromised plants represent the mean for *SO Ri* 131 and *SO Ri* 421 mutants, 3–4 independent biological replication per mutant, where each replication is a bulk of 5 independent plants. The data for the WT plants represent mean obtained from 4–8 independent biological replications, where each replication is a bulk of 5 independent plants. The metabolites data integrated by median normalization are compiled in Supplementary Table S1.

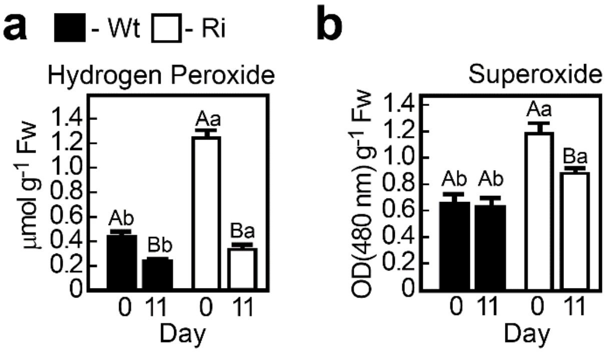

Supplementary Figure S3. Reactive oxygen species (ROS) in wild-type (WT) and SO RNA interference mutants (Ri) plants grown under normal growth conditions (0 day) and after being exposed to eleven days extended dark stress (11 day). Top leaves of WT and Ri tomato plants were used to detect hydrogen peroxide (**a**) and superoxide (**b**) in extract treated with 2 mM tungstic acid and 1 mM DPI to stop ROS generating activity in the extract. The bars are the average values ± SE (n = 3–6 individual experiments). The values denoted with different letters are significantly different according to the Turkey-Kramer HSD test (JMP 8.0 software, http://www.jmp.com/; $p < 0.05$). Different lower case letters indicate differences between SO mutant and wild-type plants within the same treatment. Different upper case letters indicate significant difference within the plant genotypes in response to treatment. The data for SO-compromised plants represent the mean for SO Ri 131 and SO Ri 421 mutants.

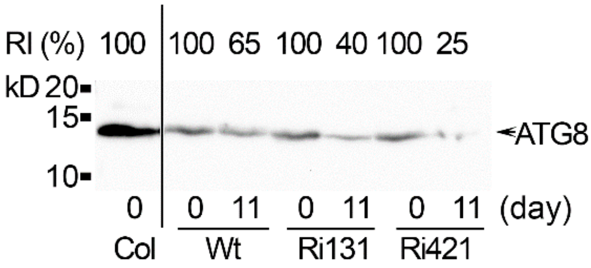

Supplementary Figure S4. The level of ATG8 protein in wild-type (Wt) and SO RNA interference mutants (Ri) plants grown under normal growth conditions (0 day) and after being exposed to eleven days extended dark stress (11 day). ATG8 protein was detected with ATG8 specific antibody. Arabidopsis Col-0 were used as positive controls.

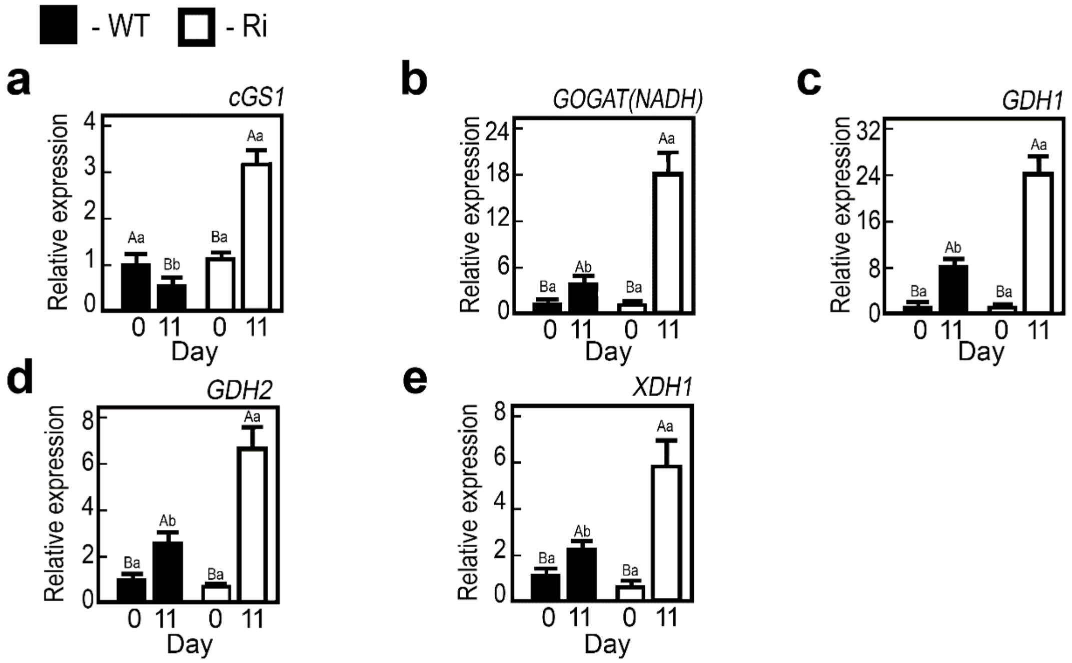

Supplementary Figure S5. The expression levels of selected nitrogen assimilation and purine catabolism genes in wild-type (WT) and SO RNA interference mutants (Ri) plants grown under normal growth conditions (0 day) and after being exposed to eleven days extended dark stress (11 day). Top leaves of WT and Ri tomato plants were used to determine the quantitative transcript expression analysis of the (**a**) Cytosolic glutamine synthase 1 (cGS1); (**b**) Glutamate synthase GOGAT (NADH); (**c**) Glutamate dehydrogenase*1 (GDH1)*; (**d**) Glutamate dehydrogenase*2 (GDH2)* and (**e**) xanthine dehydrogenase1 (*XDH1*). The relative expression after normalization to *TFIID (SGN-U329249)* is calculated by comparison with corresponding gene expression in WT plants at day 0 (set as 1.0). The values denoted with different letters are significantly different according to the Turkey-Kramer HSD test [JMP 8.0 software, http://www.jmp.com/; $p < 0.05$, ($n = 6$)]. Different lower case letters indicate differences between SO mutant and wild-type plants in the same treatment. Different upper case letters indicate significant difference within the plant genotypes in response to treatment. The data for SO-compromised plants represent the mean for SO Ri 131 and SO Ri 421 mutants.

Supplementary Table S2. Turnover of sulfur containing metabolites in wild-type (Wt) and SO RNA interference mutants (Ri) plants grown under normal growth conditions (0, day) and after being exposed to extended dark stress for eleven days (11, day) [N.B.].

**S-containing metabolites, (µmol g^{-1} Fw)**		**Wt**		**Ri**		**Degraded S-metabolites**	
		**0 day**	**11 day**	**0 day**	**11 day**	**Wt**	**Ri**
S Inorganic	Oxidized S	6.1980 Bb	10.0859 Aa	8.6343 Aa	8.8121 Ab	−3.8879 b	−0.1779 a
	Reduced S (H_2S)	0.0153 Ab	0.0133 Aa	0.0198 Aa	0.0132 Ba	0.0020 b	0.0066 a
S Organic	Total cysteine	0.3366 Ab	0.1403 Ba	0.5971 Aa	0.1397 Ba	0.1963 b	0.4574 a
	Total methionine	0.6326 Ab	0.4984 Ba	0.8858 Aa	0.4496 Ba	0.1343 b	0.4362 a
	Total glutathione	0.3381 Aa	0.2120 Bb	0.3457 Aa	0.3125 Aa	0.1260 a	0.0332 b
	SQDG	0.4184 Aa	0.3071 Ba	0.3955 Aa	0.3184 Aa	0.1112 a	0.0771 a
	acetyl-Co-A	0.0029 Ab	0.0007 Ba	0.0057 Aa	0.0012 Ba	0.0022 b	0.0045 a
	Co-A	0.0114 Ab	0.0036 Bb	0.0168 Aa	0.0073 Ba	0.0078 a	0.0095 a
	Other S-compounds	11.3289 Ba	16.4191 Aa	8.8302 Bb	17.8548 Aa	−5.0903 a	−9.0246 b
Total S		19.2821 Ba	27.6804 Aa	19.7309 Ba	27.9088 Aa	−8.3983 a	−8.1779 a
Total organic S		13.0688 Ba	17.5812 Aa	11.0768 Bb	19.0835 Aa	−4.5124 b	−8.0667 a

N.B. The sulfur containing metabolites were detected in the top leaves as an addition to the published in [14]. The contribution of other S-compounds was calculated as the difference between known S-containing metabolites to the total content of sulfur. The organic S was calculated as the difference between total S to the inorganic S (oxidized + reduced). The degraded S-metabolites were calculated as the difference between metabolite content in the unstressed plants (0d) to the content detected in plants after the dark stress (11 day). The values denoted with different letters are significantly different according to the Turkey-Kramer HSD test (JMP 8.0 software, [37]; $p < 0.05$. n = 3–10). Different lower case letters indicate differences between SO mutant and wild-type plants at the same treatment. Different upper case letters indicate significant difference within the plant genotypes in response to treatment. The data for SO-compromised plants represent the mean for SO Ri 131 and SO Ri 421 mutants.

Supplementary Table S3. Free and protein bound amino acids in wild-type (Wt) and SO RNA interference mutants (Ri) plants grown under normal growth conditions (0 day) and after being exposed to eleven days extended dark stress (11 day).

**Amino Acids ($nmol\ g^{-1}$ Fw)**	**-------------Bound Amino Acids----------------**				**-------------Free Amino Acids----------------**				**Degraded AA**	
	**WT 0 day**	**WT 11 day**	**Ri 0 day**	**Ri 11 day**	**WT 0 day**	**WT 11 day**	**Ri 0 day**	**Ri 11 day**	**WT**	**Ri**
Asp	4573 Aa	2093 Ba	5045 Aa	2429 Ba	219 Bb	945 Ab	353 Ba	1510 Aa	1754 a	1459 a
Lys	3063 Ab	1382 Ba	3422 Aa	1611 Ba	3 Bb	720 Aa	14 Ba	293 Ab	964 b	1532 a
Met	624 Ab	290 Ba	866 Aa	243 Ba	15 Ba	222 Aa	13 Ba	198 Aa	127 b	438 a
Thr	2836 Ab	1347 Ba	3226 Aa	1573 Ba	178 Bb	463 Aa	268 Aa	295 Ab	1204b	1626 a
Glu	4964 Ab	2254 Ba	5537 Aa	2628 Ba	371 Bb	1450 Aa	516 Ba	1225 Aa	1631 b	2200 a
Pro	2681 Ab	1288 Ba	3040 Aa	1486 Ba	39 Bb	31 Ab	66 Aa	73 Aa	1401 a	1547 a
Cys	314 Ab	114 Ba	579 Aa	104 Ba	16 Ba	21 Aa	20 Ba	35 Aa	195 b	460 a
Gly	4512 Ab	2209 Ba	5016 Aa	2573 Ba	210 Ab	257 Aa	566 Aa	270 Ba	2256 a	2739 a
Ser	2525 Ab	1212 Ba	2844 Aa	1411 Ba	542 Ba	945Aa	623 Ba	994 Aa	910 a	1062 a
Ala	4467 Ab	2211 Ba	5087 Aa	2530 Ba	801 Bb	334 Ab	1315 Aa	943 Aa	2723 a	2929 a
Leu	4537 Ab	2182 Ba	5075 Aa	2519 Ba	52 Ba	265 Aa	66 Ba	289 Aa	2142 a	2333 a
Val	3630 Ab	1685 Ba	4063 Aa	1988 Ba	87 Ba	407 Aa	116 Ba	377 Aa	1625 a	1814 a
Phe	2322 Ab	1158 Ba	2597 Aa	1317 Ba	38 Ba	1819 Aa	34 Ba	2225 Aa	-617 a	-911 a
Tyr	1653 Ab	787 Ba	1846 Aa	905 Ba	19 Bb	1851 Aa	31 Ba	1076 Ab	-966 b	-104 a
His	1166 Ab	555 Ba	1349 Aa	655 Ba	20 Ba	227 Aa	13 Aa	49 Ab	404 b	658 a
Total AA	43825 Ab	20774 Ba	49700 Aa	23972 Ba	2692 Bb	9957 Aa	4014 Ba	9852 Aa	15835 b	19782 a

Asp—aspartate; Lys—lysine; Met—methionine; Thr—threonine; Glu—glutamate; Pro—proline; Cys—cysteine; Gly—glycine; Ser—serine; Ala—alanine; Leu—leucine; Val—valine; Phe—phenylalanine; Tyr—tyrosine; His—histidine. Degraded Amino acid/s were calculated as the difference between bound at 0 day and 11 day minus the difference between free amino acid/s at 0 day and 11 day. The values denoted with different letters are significantly different according to the Turkey-Kramer HSD test (JMP 8.0 software, http://www.jmp.com/; $p < 0.05$, $n = 3$). Different lower case letters indicate differences between SO mutant and wild-type plants at the same treatment. Different upper case letters indicate significant difference within the plant genotypes in response to treatment. The data for SO-compromised plants represent the mean for SO Ri 131 and SO Ri 421 mutants.

Supplementary Table S4. List of primers used for quantitative real-time PCR (Tomato, *Lecopersicum esculentum Mill*).

Transcript, Accession Number	Primer's Sequence
ACTIN Tom41; U60480	Fw-CATGCCATTCTCCGTCTTGA<br>Rw-CGCTCGGTCAGGATCTTCAT
EF 1-α (Elongation factor 1-alpha); SGN-U196120	Fw-CCTACTTGAGGCTCTTGACCAGATT<br>Rw-AAAAGTGACAACCATACCAGGCTTAAT
GOGAT(NADH) Glutamate synthase, NADH/NADPH; SGN-U575483	Fw-TTCTGAGAGAACCGGGAGAAGAGTT<br>Rw-CAATTTTGTCGGTCTTCATGTTGG
XDH1 (Solanum lycopersicum xanthine dehydrogenase 1-like, LOC101252457)	Fw-TCATCTACCCAGGCTCCGCAGAAG<br>Rw-ACAGCAGCAGCAAGCATAGCAGAC
GDH1 (Solanum lycopersicum Glutamate dehydrogenase 2, SGN-U578318)	Fw-GTGGTAACTGGAAAACCTGTTGATCTC<br>Rw-GAACCAACATTACCAAATCCCTGTATAA
GDH2 (Solanum lycopersicum Glutamate dehydrogenase 2, SGN-U574592)	Fw-TCCTTTCAGAGAAATTAAGGTGGAATG<br>Rw-CATTTACCTCATCTGGGTCAACCTC
TFIID; SGN-U329249	Fw-ATAGTCCCTACGCTCCAGAATATTGTCTC<br>Rw-CTCCAGTACAAACCATTTTCCCAGAAG
cGS1 Cytosolic glutamine synthetase; SGN-U577193	Fw-CAGGACTCTCCCTGGTCCAGTTAC<br>Rw-AGTATAGGCATCACACATGACCAAGAT
clGS1 Chloroplast glutamine synthetase; SGN-U578728	Fw-CCGGACCTCAGGGTCCTTACTACT<br>Rw-GGACCTACTTGAAATTCCCACTGTCh
WRKY WRKY transcription factor 2d-1; SGN-U563810	Fw-GATGGCTTTTGAGTTAACAGGACAGA<br>Rw-CAAATTTACATACACACCCCTCAACTG

Supplementary Table S5. The effect of genotype and dark stress on the ratios between the total (protein bound and free amino acids) S-amino acids (Cys and Met) and the non-S total detected amino acids profile in wild-type (WT) and SO RNA interference mutants (Ri) plants.

	WT		Ri	
Time in Dark, day	0	11	0	11
* Non S total detected AA (NSAA), (μmol g^{-1} Fw)	45.88	30.22	52.84	33.38
* Total S AA (SAA) (μmol g^{-1} Fw)	0.97	0.64	1.49	0.58
SAA/NSAA (×100, %)	2.1 Ab	2.1 Aa	2.8 Aa	1.7 Bb

The values denoted with different letters are significantly different according to the Turkey-Kramer HSD test (JMP 8.0 software, http://www.jmp.com/; $p < 0.05$). Different lower case letters indicate significant differences between wild-type and SO mutant plants. Different upper case letters indicate significant differences within the plant genotypes in response to treatment. The data for SO-compromised plants represent the mean for SO Ri 131 and SO Ri 421 mutants. SAA/NSAA (×100, %) indicates the ratio in percentage of the total detected sulfur containing amino acids to the total detected non sulfur amino acids. * Data from Supplementary Table S3 and Supplementary Figure S1 in Reference [14].
